# Supplementary material for: The Inhibitory Effect of Propylene Glycol Alginate Sodium Sulfate on Fibroblast Growth Factor 2-Mediated Angiogenesis and Invasion in Murine Melanoma B16-F10 Cells In Vitro
Source: Mar Drugs. 2019 Apr 29;17(5):257. doi: 10.3390/md17050257 (PMC6562581; doi:10.3390/md17050257)
Supplement: Supplementary file 1 [file marinedrugs-17-00257-s001.pdf]

The Inhibitory Effect of Propylene Glycol Alginate Sodium Sulfate on FGF2-Mediated Angiogenesis and Invasion in Murine Melanoma B16-F10 Cells In Vitro

He Ma[1], Peiju Qiu[1,2,3,4]*, Huixin Xu[1], Ximing Xu[1,2,3,4], Meng Xin[1,2,3,4], Yanyan Chu[1,2,3,4], Huashi Guan[1,2,3,4], Chunxia Li[1,3] and Jinbo Yang [1,2,3,4]*

[1] *Key Laboratory of Marine Drugs of Ministry of Education, Shandong Provincial Key laboratory of Glycoscience and Glycotechnology, School of Medicine and Pharmacy, Ocean University of China, Qingdao 266003, China.*

[2] *Innovation Center for Marine Drug Screening & Evaluation, Pilot National Laboratory for Marine Science and Technology (Qingdao), Qingdao 266237, China.*

[3] *Laboratory for Marine Drugs and Bioproducts of Pilot National Laboratory for Marine Science and Technology (Qingdao), Qingdao 266237, China.*

[4] *Marine Biomedical Research Institute of Qingdao, Qingdao 266071, P.R. China.*

***Corresponding authors:** Ocean University of China, School of Medicine and Pharmacy, 5 Yushan Road, Qingdao 266003, Shandong Province, China.

Tel.+86 532 85906859, +86 532 82032030

E-mail: peijuqiu@ouc.edu.cn (Peiju Qiu)

yangjb@ouc.edu.cn (Jinbo Yang)

Supplementary Materials

Method

1. HUVEC cell migration assay

The rate of HUVEC migration was monitored in real-time with the xCELLigence system (CIM plates). The upper chamber of the 16 CIM plates was coated with 1 μg/μL of fibronectin. A total of 40,000 HUVECs were seeded into each well of the upper chamber in serum-free media. Fresh culture medium contained 10% FBS and varying concentrations of PSS or heparin (50, 100 μg/mL) was added to each well of the lower chamber. The CIM plates was left in an incubator for 1 h to allow cell attachment. The impedance value of each well was automatically monitored by the xCELLigence system for a period of 70 h and is expressed as a CI value.

Results

Table 1. Analysis of the affinity between PSS and VEGF165.

Protein	PSS	Heparin
**VEGF165**	1.78×10^{-4} M	8.09×10^{-7} M

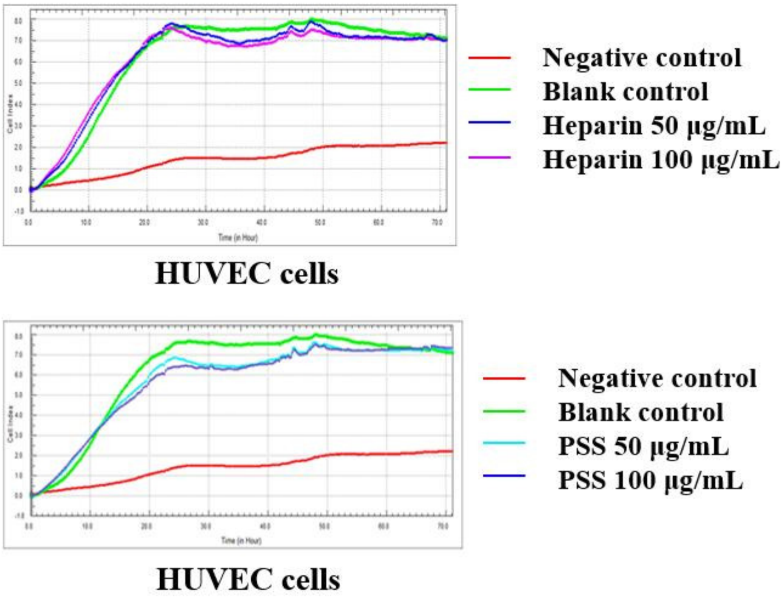

Supplementary Figure 1. The effect of PSS on the migration of HUVEC cells. HUVECs (4×10^4 cells/well) were seeded in 16 CIM plates, treated with various concentrations of PSS, and allowed to migrate for 70 h. The migrating cells were monitored by xCELLigence Real-Time Cell Analyzer. The results are from three independent experiments.

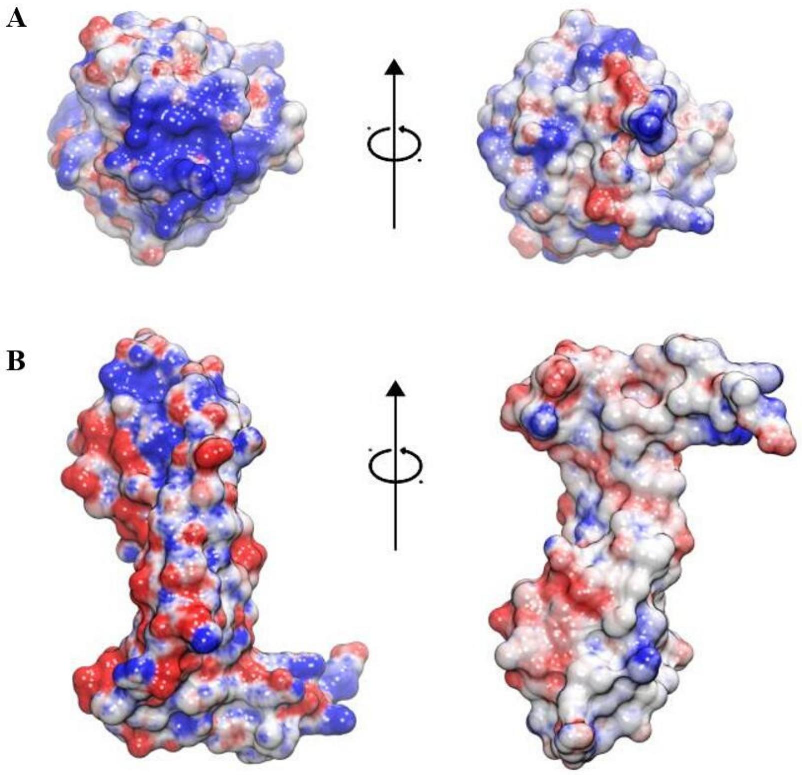

Supplementary Figure 2. The electrostatic potential surface of FGF2 (A) and VEGF165 (B). The positive charge and negative charge are colored in blue and red respectively. The structures of VEGF165 and FGF2 were extracted from x-ray crystal structures with PDBID# 3V2A and 1CVS respectively. CHARMM partial charge was assigned to the two structures at pH7.0 by PDB2PQR program [1], then electrostatical potential was calculated by APBS program [2]. Representation was prepared by VMD program [3].

References

1. Dolinsky, T. J.; Nielsen, J. E.; McCammon, J. A.; Baker, N. A., PDB2PQR: an automated pipeline for the setup of Poisson-Boltzmann electrostatics calculations. *Nucleic Acids Res.* **2004,** 32, (Web Server issue), W665-7.
2. Baker, N. A.; Sept, D.; Joseph, S.; Holst, M. J.; McCammon, J. A., Electrostatics of nanosystems: application to microtubules and the ribosome. *Proc Natl Acad Sci U S A.* **2001,** 98, (18), 10037-41.
3. Humphrey, W.; Dalke, A.; Schulten, K., "VMD - Visual Molecular Dynamics", *J Mol Graph.* **1996**, 14, (1), 33-8, 27-8.
